# Refinement of protein‐protein complexes in contact map space with metadynamics simulations

**DOI:** 10.1002/prot.25612

**Published:** 2018-10-30

**Authors:** Erik Pfeiffenberger, Paul A. Bates

**Affiliations:** ^1^ Biomolecular Modelling Laboratory The Francis Crick Institute London United Kingdom

**Keywords:** metadynamics simulations, protein‐protein complexes, refinement, scoring functions

## Abstract

Accurate protein‐protein complex prediction, to atomic detail, is a challenging problem. For flexible docking cases, current state‐of‐the‐art docking methods are limited in their ability to exhaustively search the high dimensionality of the problem space. In this study, to obtain more accurate models, an investigation into the local optimization of initial docked solutions is presented with respect to a reference crystal structure. We show how physics‐based refinement of protein‐protein complexes in contact map space (CMS), within a metadynamics protocol, can be performed. The method uses 5 times replicated 10 ns simulations for sampling and ranks the generated conformational snapshots with ZRANK to identify an ensemble of *n* snapshots for final model building. Furthermore, we investigated whether the reconstructed free energy surface (FES), or a combination of both FES and ZRANK, referred to as CS_*α*_, can help to reduce snapshot ranking error.

## 1 INTRODUCTION

The vast majority of all proteins are involved in assemblies where they form stable complexes with one or more partners, or more often, form transient interactions with a large number of different partners. Resolving the three‐dimensional description of these interactions, to atomic detail, is crucial for understanding biological function.^1^, ^2^ However, despite the ever‐increasing number of new structures,^3^ the number of resolved structures of protein‐protein complexes in the Protein Data Bank (PDB)^4^ remains limited. This relative paucity of protein‐protein complexes, particularly to atomic resolution, limits our understanding of the workings of protein‐protein interactions. Thus, accurate in silico predictions of protein‐protein interactions seem to be the only viable option to complete the missing links within structure‐based interaction networks and to fully elucidate functional relationships.^5^

Several protein‐protein docking approaches have been developed to predict the three‐dimensional interaction of proteins, which can be broadly grouped into rigid body^6^, ^7^, ^8^, ^9^, ^10^, ^11^, ^12^, ^13^ and flexible docking^14^, ^15^, ^16^, ^17^, ^18^ methods. To model transitions from unbound to bound states, the former considers only translational and rotational search space, whereas the latter also incorporates conformational flexibility into the docking process. Rigid body docking, where the conformations of the unbound complex components are equal to the bound, is considered a solved problem;^19^ many highly optimized algorithms based on fast‐Fourier transformation (FFT) techniques and geometrical hashing are utilized to obtain accurate models. However, these methods fail to generate high‐accuracy models when the proteins undergo complex conformational changes from the unbound to bound form.

To model side‐chain and backbone rearrangements, a high number of degrees of freedom have to be considered; therefore, heuristic optimization algorithms are required to search the solution space efficiently. The CAPRI‐experiments^20^ (Critical Assessment of PRediction of Interactions) have shown that heuristic methods are often able to find solutions with acceptable quality. Nevertheless, finding medium or high‐quality solutions still remains challenging.^21^, ^22^, ^23^, ^24^ A solution to this problem is so called refinement methods, which perform a local optimization on a docked solution in order to obtain a higher quality model. Physics‐based refinement methods, using standard algorithms from molecular dynamics, have shown anecdotal success of improving docking solutions.^25^ However, the computational cost of simulating long enough time scales to escape local minima has often been a limiting factor.

In this study, to perform more directed sampling, a method is presented that exploits a so‐called contact map space (CMS). The CMS is constructed from the observed residue‐residue contacts at the interface between a receptor and a ligand from one initial docked solution or an ensemble. To bias sampling of the binding funnel, the CMS is used as a collective variable (CV) in a metadynamics simulation. Furthermore, our work does not only investigate the sampling aspect of refinement but also how improved snapshots can be identified from the trajectory data and be used for generating a final refined model. Our analysis shows that the most reliable snapshot selection strategy for final model generation is to generically use the empirical scoring function ZRANK^26^ when the refinement category is not known, that is, from a wide variety of starting model qualities; acceptable, medium or high. Nevertheless, the mixed energy function, CS_*α*_, that takes account of free energy changes, shows utility when refining from models of acceptable quality.

## 2 MATERIALS AND METHODS

### 2.1 Method overview

**Figure 1:**
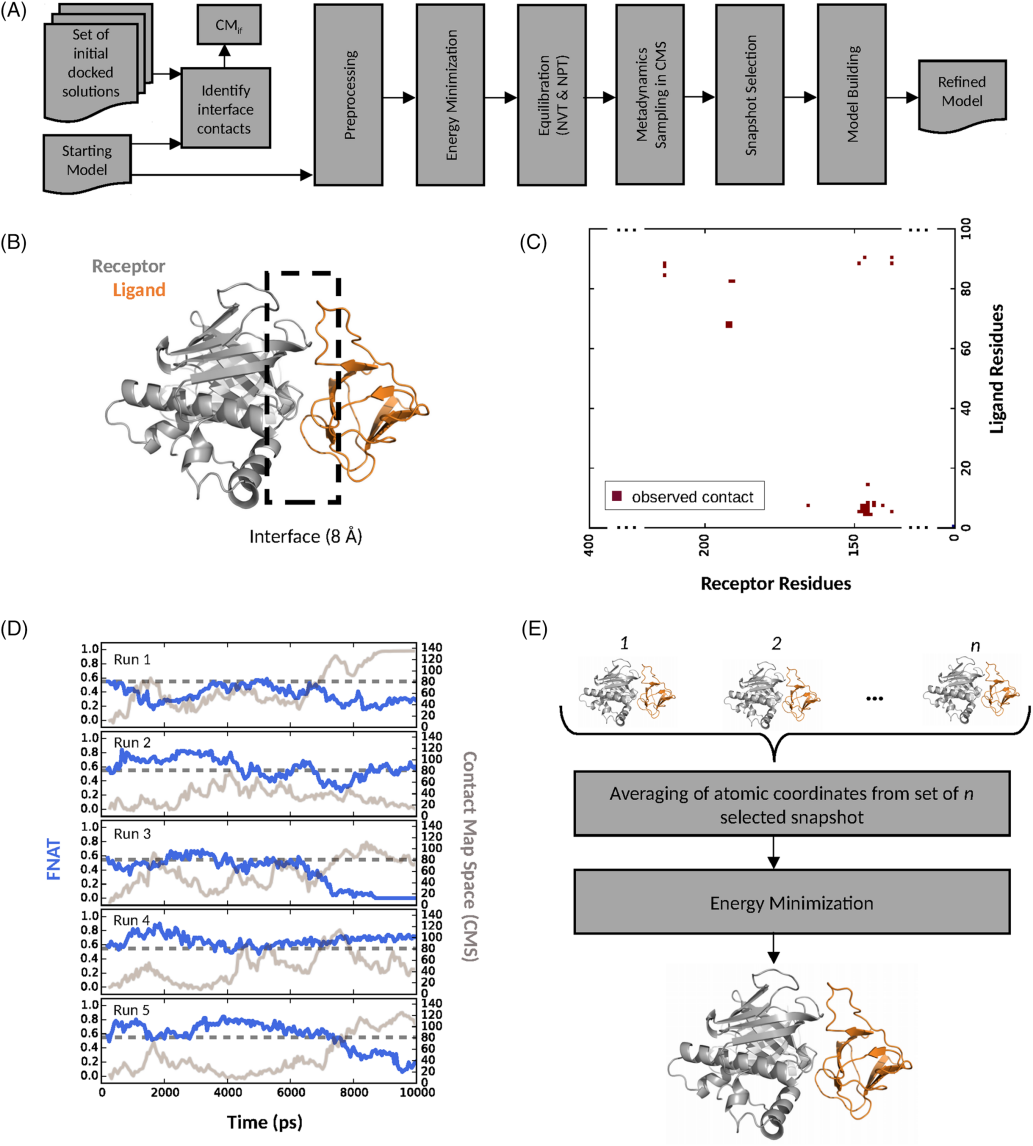
Method overview. (A) From a set of docked solutions and the starting model for refinement, interface contacts between the receptor and ligand are identified. The starting model is preprocessed by modeling possible missing atoms and residues, energy minimized and equilibrated. The sampling in CMS is performed with replicated metadynamics simulations. The generated snapshots are scored and the best *n* is selected to generate the final refined model. Plots B‐E exemplify this for target T39. (B) Schematic representation of the interface definition which includes residues which are within 8 Å of the receptor‐ligand. (C) Schematic representation of the contact map (CM_*if*_) resulting from the residue‐residue contacts at the receptor‐ligand interface. The number of observed contacts comes from the ensemble of docked solutions. (D) Example of a five times replicated sampling run of a target with metadynamics where the CV describes the CMS. The blue line represents the FNAT as a function of simulation time, the gray line the CMS as a function of simulation time and the dotted dark gray line is the starting model FNAT. (E) Selection of the best *n* scoring snapshots from the trajectories. The final model is an average of all snapshots by averaging the Cartesian coordinates of each atom followed by a two step energy minimization of the structure

The overall flow of the method is shown in Figure 1A, and a graphical overview of our refinement method is shown in Figures 1B‐E takes one of our case studies as a representative example, that of an acceptable docking pose for target T39.

Starting with a protein‐protein model docking pose, within or on the edge of the native binding funnel, and an optional set of additional docked solutions in close proximity to it (taken from the dataset described below), an improved model complex is typically generated. From this initial starting ensemble, interface contacts are identified (see Figures 1B,C) and denoted as the interface contact map (CM_*if*_). The starting model is then prepared for the metadynamics simulation in CMS by modeling missing atoms with SCWRL^27^ and missing segments with Loopy.^28^ A multistep energy minimization phase, followed by an equilibration phase, is performed to relax the starting model prior to production sampling, see simulation setup described below. During production sampling, to enhance sampling of relevant unbound to bound transitions, the CM_*if*_ map is used in a metadynamics simulation at the docked interface. To sample a sufficient degree of conformational space, 5 times replicated sampling runs are performed, see Figure 1D. Subsequently, the snapshots of the resulting trajectories are scored and ranked. From this set, the best *n* is selected to generate the final refined model, which is obtained by averaging the equivalent atomic Cartesian coordinates for all selected frames. To resolve small nonphysical perturbations, for example minor side‐chain clashes, the averaged structure is subject to further energy minimization, see Figure 1E. This methodology of averaging equivalent atomic coordinates from a selection of high scoring snapshots is motivated by a refinement‐method based on molecular dynamics for protein‐monomer models.^29^

### 2.2 Data set

The protein‐protein refinement method was benchmarked on 23 cases, using 11 targets, from the score_set data set^30^ of the CAPRI scoring experiment. The data set consists of decoys of varying quality (high, medium, acceptable, and incorrect), as contributed by all participating docking groups of the CAPRI blind docking trials; thereby, representing models generated by a wide‐range of docking methodologies. Targets containing more than one chain for receptor or ligand (T37 and T50) and targets without any acceptable, medium or high quality solutions (T36 and T38) were removed from the benchmark set (see Table 1 for the full list). The structure chosen to represent the quality category of a target was the centroid element, a calculation based on ligand root mean square deviation (LRMSD) for all models belonging to that category. Table 1 gives an overview of all starting models with their initial model quality metrics. The produced refinement trajectories of these targets can be downloaded from https://zenodo.org/record/1217537.

**Table 1:** CAPRI starting model quality

TR	SMQ	FNAT	IRMSD (Å)	LRMSD (Å)
T29	Acc	0.45	3.41	6.98
T29	Med	0.53	2.75	5.21
T29	Hig	0.82	1.82	3.83
T30	Acc	0.2	6.12	13.13
T32	Acc	0.36	2.77	8.08
T32	Med	0.49	1.96	6.57
T35	Acc	0.15	5.09	13.3
T39	Acc	0.55	2.31	7.51
T39	Med	0.78	1.32	3.65
T40	Acc	0.63	2.58	6.84
T40	Med	0.8	2.16	4.27
T40	Hig	0.8	1.03	4.32
T41	Acc	0.49	2.63	6.97
T41	Med	0.65	1.38	3.4
T41	Hig	0.78	0.8	2.48
T46	Acc	0.49	3.75	10.57
T47	Acc	0.54	2.56	5.7
T47	Med	0.79	1.32	2.84
T47	Hig	0.85	0.99	1.59
T53	Acc	0.19	5.67	13.09
T53	Med	0.48	5.7	9.62
T54	Acc	0.41	3.94	7.53
T54	Med	0.5	2.7	4.76

The FNAT, IRMSD, and LRMSD to the reference crystal structure for 23 different starting models, and from 11 different protein targets, are shown. The column SMQ describes the CAPRI starting model quality as assigned in the score_set data set with the three classes acceptable (acc), medium (med), and high (hig).

### 2.3 Definition of the contact map space

The CMS, which is a scalar value, for each protein‐protein complex describes the interface contacts of residue pairs between the designated receptor and ligand protein. To qualify as a contact, the distance between the C*α* atoms of the residue pairs has to be below 8 Å, see Figure 1B. The mathematical definition of the CMS for complex *R* is given by:^31^

(1) $$\mathrm{CMS}\left(R\right)=\sum_{\gamma \in {\mathrm{CM}}_{\text{if}}}{\left(D_{\gamma }\left(R\right)-D_{\gamma }\left(R_{\mathrm{ref}}\right)\right)}^{2}$$

and

(2) $$D_{\gamma }\left(R\right)=\frac{1-{\left(r_{\gamma }/r_{\gamma }^{0}\right)}^{n}}{1-{\left(r_{\gamma }/r_{\gamma }^{0}\right)}^{m}},$$

where *CM*_*if*_ is the contact map (CM) that contains the interface contacts between the receptor and ligand, see Figure 1C. The value range for the CMS can vary from target to target, depending on the CM_*if*_ definition. The sigmoid distance function *D*_*γ*_(*R*) quantifies the formation of a contact *γ* in structure *R*, where *r*_*γ*_ is the contact distance in structure *R* and $r_{\gamma }^{0}$ is the contact distance in reference structure *R*_ref_. If *r*_*γ*_ and $r_{\gamma }^{0}$ are the same, the distance *D*_*γ*_ is set to 0.6. Here, *R*_ref_ describes a set of models of a target, that is, the docked solutions of the score_set that have the same starting model quality as the selected starting model. Variables *n* and *m* are constant and set to *n* = 6 and *m* = 10.

### 2.4 Simulation setup

All starting models were checked for missing residues and atoms, and where necessary completed with the program Loopy^28^ and SCRWL;^27^ see Supporting Information for details. The system was solvated in a cubic simulation box, with a buffer of 12 Å, using the explicit solvent model SPC/E^32^ and with the overall charge neutralized by Na^+^ and Cl^−^ ions at a concentration of 0.15 mol/L. The energy minimization was performed with GROMACS 4.6^33^ and consisted of the following three steps: (1) steepest‐descent energy minimization with 50 000 steps and a step‐size of 0.01; (2) conjugate gradient‐based minimization with 500 000 steps and one steepest‐descent step every 1000 steps; (3) a second round of steepest‐descent minimization for 50 000 steps. Each minimization step is stopped early when the maximum force is <100 kJ mol^‐1^nm^‐1^. Subsequently, the equilibration of the system, using GROMACS 4.6, followed a two‐step protocol: the first phase consisted of a 100 ps long NVT equilibration, where an increase of the temperature with V‐rescale^34^ from 0 K to 300 K was performed; in the second step of the equilibration, the pressure of the system was increased to 1 bar, with Parrinello Rahman pressure coupling,^35^ for a simulation time of 300 ps. During NVT and NPT, all heavy atoms were subject to position restraints with a force of 1000 kJ mol^‐1^nm^‐1^. A short‐range Coulomb and van der Waals cut‐off distance of 1 nm was used. For long range electrostatic calculations, the Particle Mesh Ewald method with a cubic interpolation of 4 and a grid spacing of 0.16 nm was used.

The production run with metadynamics in CMS, as defined in Equation (1), was performed with PLUMED2^36^ and GROMACS 4.6. The same values for van der Waals and Coulomb cutoffs were used, along with Parrinello Rahman pressure coupling and V‐rescale for temperature coupling. The Gaussian addition, to bias the potential along the CV, was deposited every 2 ps, with *σ* = 0.5, and a bias factor of 10 and an initial height of 5 kJ mol^‐1^. The *σ* value describes the width of the addition to the potential, and the initial height in kJ mol^‐1^ the quantity. The bias factor expresses the ratio between the temperature of the CV and the system temperature.^37^, ^38^ The sampling was performed for 10 ns with a Δ*t*= 2 fs. A total of five replicated production runs were performed for each refinement case analyzed (see Figure 1D). Our scalar collective variable, CMS, gently guides the refinement but does not over constrain refinement space. However, this does sometimes lead to some variability in the quality metrics between each run, see Figure 1D, where maximum FNAT from run 1 is 0.6 and for run 2 is 0.8.

### 2.5 Definition of the scoring function *CS*_*α*_

The new scoring function, *CS*_*α*_, combines the free energy surface (FES), reconstructed from metadynamics simulations, with the ZRANK scoring function (a weighted additive scoring function of detailed van der Waals, electrostatic and desolation terms), and is defined as follows:

(3) $${\mathrm{CS}}_{\alpha }=\alpha {\text{ZRANK}}_{\eta }+\left(1-\alpha \right){\mathrm{FES}}_{\eta },$$

where ZRANK_*η*_ and FES_*η*_ are the 0‐1 normalized energies. The parameter *α* is a weighting factor that ranges from 0 to 1. Therefore, an *α*‐value of 1 means that only ZRANK_*η*_ is considered for the scoring and a value of 0 means that only the FES_*η*_ is considered.

The correct rank for a set of snapshots *S*, for each target, is given by the ascending order of their LRMSD to the reference crystal structure. This sorted list of snapshots is defined as *sort*_*lrmsd*_(*S*). Furthermore, the maximum rank is capped such that

(4) $$\mathrm{rank}\left(S\right)=\left\{\begin{array}{ll} i, & \text{if}\;i\leq \max\\ \max, & \text{otherwise} \end{array}\right.$$

where *max* is the threshold used when *i* > *max*. Applying these two functions to *s* gives the reference ranking *R* =  *rank* (*sort*_*lrmsd*_(*S*)). The rank assignment based on function *CS*_*α*_ is the descending order of their scores and the ranks produced by this function is denoted as *C* =  *rank* (*sort*_*cs*_(*S*)). Following this notation, the rank for snapshot *i* is retrieved by *R*_*i*_ and *C*_*i*_, respectively. The ranking error *ε* produced by *CS*_*α*_ can now be quantified with

(5) $$\varepsilon =\sum_{\mathrm{TR}}\sum_{i=0}^{C_{:n}}r_{i},$$

where *n* is the number of snapshots that are used for ranking, *C*_:*n*_ defines the subset of ranks from the 1st to the *n*th snapshot, and *TR* is the set of targets. In an additional step, *ε* is normalized to

(6) $$\varepsilon_{\eta }=\frac{\varepsilon -{\mathrm{rank}}_{\min}}{{\mathrm{rank}}_{\max}-{\mathrm{rank}}_{\min}},$$

where *rank*_*min*_ =  ∣ *TR* ∣ ((*n*(*n* + 1))/2) and *rank*_*max*_ =  ∣ *TR* ∣ (*max* + 1)*n*.

### 2.6 Model building

The generic model building protocol proposed is based on the best *n* ranked snapshots from *ZRANK*. The final model is computed by averaging each atom's coordinates from the *n* selected snapshots from a target's trajectory.^29^ Snapshots, with a Δ*t*= 50 ps, are considered for model building, that is, where each snapshot is spaced in an interval of 50 ps. Energy minimization of the averaged model, with steepest‐descent and 50 000 steps, was performed to resolve nonphysical conformations. In the following text, this model building strategy is referred to as AZRANK.

### 2.7 Model assessment measures

Model quality is assessed by LRMSD, interface root mean square deviation (IRMSD) and fraction of native contacts (FNAT). The calculation of these assessment measures follows the formulation described for the CAPRI blind docking trials.^21^, ^22^ Below we outline these calculations, pointing out any variation from the original formulation. The LRMSD quantifies the translational, rotational and conformational deviation of the predicted ligand model to the reference model. The RMSD between predicted ligand position and reference ligand position is computed after optimally superimposing the receptor of the predicted complex to the reference model. The superimposition as well as the RMSD calculation is based on C*α*‐atoms. The IRMSD describes the conformational difference at the receptor‐ligand interface between the predicted model and the reference model. The set of interface atoms are given by observed residue‐residue contact in the reference crystal structure. Here, a residue in the ligand is in contact with the residue in the receptor if any of their atoms has a distance <10 Å. The IRMSD calculation is based on C*α*‐atoms only and interface atoms of the predicted and reference model are first optimally superimposed. The FNAT quantifies the relative number of correctly predicted residue‐residue contacts between a receptor and a ligand as observed in the reference crystal structure, where a residue‐residue contact is defined as any of their atoms within a distance less than 5 Å. FNAT values can range from 0, that is, no correctly predicted contact, to 1, all contacts are correctly predicted. From the above three metrics, a CAPRI quality classification of each refined protein‐protein docked complex was performed and categorized in the order of increasing accuracy to the reference crystal structure as incorrect, acceptable, medium, and high. The assignment of these quality classes for the starting model solutions, in the score_set data set, was directly taken from their annotation.

## 3 RESULTS

### 3.1 Refinement success of the model building strategy AZRANK

Here, we analyze how often our optimum refinement strategy, AZRANK, which takes as the final refinement model an equivalent atom coordinate average of the 14 best snapshots selected from 5 metadynamics simulation runs, improves the starting model docked pose relative to the three metrics; FNAT, LRMSD, and IRMSD. The number of 14 snapshots was determined by extensive empirical testing, the Supporting Information text and Supporting Information Figures S1 and S2 provide more information about this procedure. In addition, an interesting question to ask of our refinement method is how often the final model is as good in quality, if not better, than the absolute best snapshot, should it have been selected. This provides a measure of how good the energy function, in this analysis just ZRANK, can select the best, or at least the better‐quality, snapshots. Results to the analysis are shown in Table 2. Here, for each refinement category, consisting of the target number and starting model quality (acceptable, medium or high), changes in the three model assessment metrics is reported.

**Table 2:** Complex model quality after refinement

		Build model with n = 14			Best snapshot		
TR	SMQ	ΔFNAT	ΔLRMSD	ΔIRMSD	ΔFNAT	ΔLRMSD	ΔIRMSD
T29	Acc	**0.08**	**−1.66**	**−0.90**	**0.24**	**−4.25**	**−1.59**
T29	Med	−0.04	1.61	0.70	**0.16**	**−2.35**	**−0.38**
T29	Hig	−0.04	**−0.10**	**−0.37**	0.00	**−1.15**	**−0.22**
T30	Acc	**0.09**	1.75	**−0.25**	**0.25**	**−3.55**	**−0.75**
T32	Acc	**0.22**	**−4.75**	**−1.13**	**0.24**	**−4.93**	**−1.53**
T32	Med	−0.01	**−3.25**	**−0.43**	**0.16**	**−3.48**	**−0.84**
T35	Acc	−0.06	0.10	0.35	**0.02**	**−5.04**	**−0.87**
T39	Acc	**0.24**	**−5.92**	**−1.28**	**0.35**	**−6.03**	**−1.76**
T39	Med	**0.16**	**−1.5**	**−0.10**	**0.18**	**−2.35**	**−0.50**
T40	Acc	0.00	**−1.29**	**−0.60**	**0.11**	**−4.62**	**−0.70**
T40	Med	−0.05	**−0.27**	**−0.24**	**0.02**	**−2.20**	**−0.62**
T40	Hig	−0.08	4.58	1.30	**0.13**	**−2.24**	**−0.15**
T41	Acc	−0.13	1.12	1.75	**0.04**	**−3.57**	**−0.42**
T41	Med	−0.25	1.50	1.64	**0.07**	**−1.13**	0.12
T41	Hig	−0.31	1.96	1.54	**0.03**	**−0.92**	0.09
T46	Acc	−0.03	0.03	**−0.30**	**0.01**	**−3.00**	**−0.08**
T47	Acc	**0.06**	0.91	**−0.21**	**0.17**	**−2.76**	**−1.12**
T47	Med	−0.13	2.87	0.77	**0.02**	**−1.34**	**−0.22**
T47	Hig	−0.10	1.93	0.46	**0.06**	**−0.07**	**−0.01**
T53	Acc	**0.17**	**−0.69**	0.97	**0.29**	**−2.45**	**−0.84**
T53	Med	**0.04**	0.92	**−1.45**	**0.33**	**−3.20**	**−1.01**
T54	Acc	**0.09**	**−1.78**	**−1.21**	**0.19**	**−3.22**	**−1.64**
T54	Med	**0.00**	**−0.13**	**−0.27**	**0.09**	**−1.39**	**−0.37**

Results from 11 different target complexes (TR) with different CAPRI starting model qualities (SMQ) acceptable (acc), medium (med), and high (hig). Refinement performance is shown for AZRANK with n = 14 snapshots and the theoretical best improvement by selecting the best quality snapshot. The metrics ΔFNAT, ΔLRMSD (Å), and ΔIRMSD (Å) show the relative change to the starting model values, where bold text indicates an improvement over the initial model quality.

Overall, the refinement with model building strategy AZRANK was most successful for starting models with acceptable quality; here the FNAT, LRMSD and IRMSD could be improved for 7, 6, and 8 out of 11 targets, respectively. For starting models with medium quality, the FNAT, LRMSD, and IRMSD could be improved 2, 4, and 5 out of 8 targets, respectively. For the four high quality examples in the test‐set the FNAT, LRMSD, and IRMSD were improved 0, 1, and 2 times, respectively. The theoretical best refinement success, if the best snapshot would have been selected as the final model, yields good results for all three starting model quality classes (see Table 2. The FNAT, LRMSD, and IRMSD could be improved for all acceptable quality models. The sampling for medium quality starting models failed only for target T41 (where the IRMSD decreased slightly by 0.12 Å), whereas all other metrics, for all other targets, could be improved. Similarly, for the case of starting models with high quality, a decrease in quality after refinement sampling was only observed for target T41, where the IRMSD decreased by 0.09 Å.

**Figure 2:**
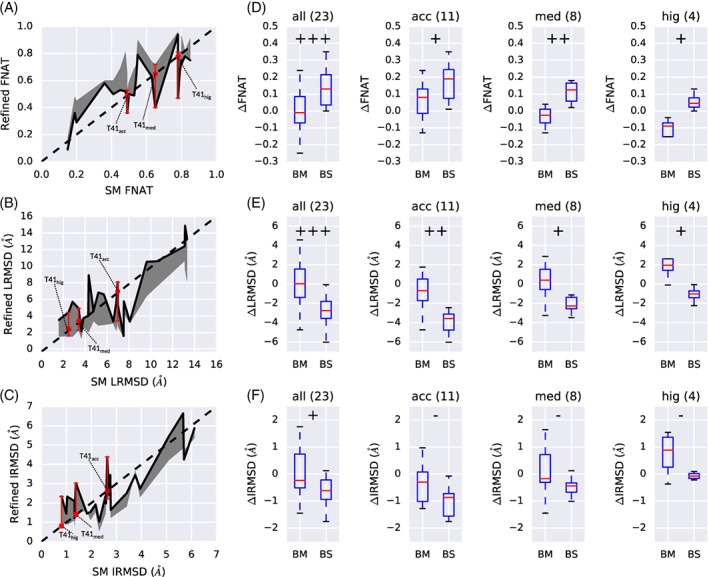
Complex refinement result overview. Plots A‐C show the results for all benchmark cases. (A) Starting model (SM) FNAT vs refined FNAT. (B) Starting model (SM) LRMSD vs refined LRMSD. (C) Starting model (SM) IRMSD vs refined IRMSD. For plots (A‐C), the black line indicates the change based on build model with AZRANK, and the gray area visualizes the gap between best snapshot and build model. (D‐F) Split down of refinement results with respect to starting model quality all, acceptable (acc), medium (med), and high (hig). The number in brackets indicates the number of refined models in that category. The symbols +++, ++, +, and ‐ indicate significance level between build model (BM) and best snapshot (BS) at *P*‐value <.001, *P*‐value <.01, *P*‐value <.05, and *P*‐value ≥.05, respectively

Figure 2A shows the success at improving the FNAT as a function of starting model (SM) FNAT. The analysis of our method shows that for a large range of initial values, that is, 0.2‐0.6, improved quality models could be generated with the model building strategy AZRANK and 14 snapshots. For SMs with higher FNAT values (0.6‐0.8), the success of AZRANK is less pronounced. However, this is mainly due to target T41, which produced negative refinement results for all three model quality categories (see red bars in Figure 2A). The analysis of the refinement performance as a function of starting model LRMSD shows good results for medium LRMSD values in the range from 6 to 9 Å. However, starting from lower LRMSD values (1.5‐6 Å) produces a mixed set of results, with cases that could yield improved models but with some cases that could not. Refinements of models for higher starting LRMSD values (ie, >9 Å) produce snapshots with large improvements, however, model building based on AZRANK is less able to identify these and improvements in LRMSD are small or not possible. Refinement performance as a function of SM IRMSD is shown in Figure 2C. The generation of improved IRMSD snapshots and models with AZRANK is most successful in the SM IRMSD range from 1.8 to 4.5 Å. Lower IRMSD values from 1 to 1.8 Å show no improvement; the generated models with AZRANK could not produce improvements and even the sampled best snapshot for these targets had only minor improvements. Refinement on models with SM IRMSD >4.5 Å produce improved sampled snapshots and to some extent improved build models with AZRANK.

The level of refinement success is enhanced if the best snapshot could have been selected as the final model, see last three columns of Table 2, yielding a 100% success rate (if an improvement in at least one metric is counted), with notable improvements over all three starting model quality classes (see Table 2). Indeed, a closer look at the differences of the extent of improvement between the best sampled snapshot and the built model with AZRANK shows the inadequacy of the scoring function ZRANK to identify the highest quality snapshots from the trajectory. The difference for the three metrics FNAT, LRMSD, and IRMSD is significant, that is, *P* value <.05, considering all 23 test‐cases (see Figure 2D‐F).

### 3.2 Refinement success as a function of simulation time

**Figure 3:**
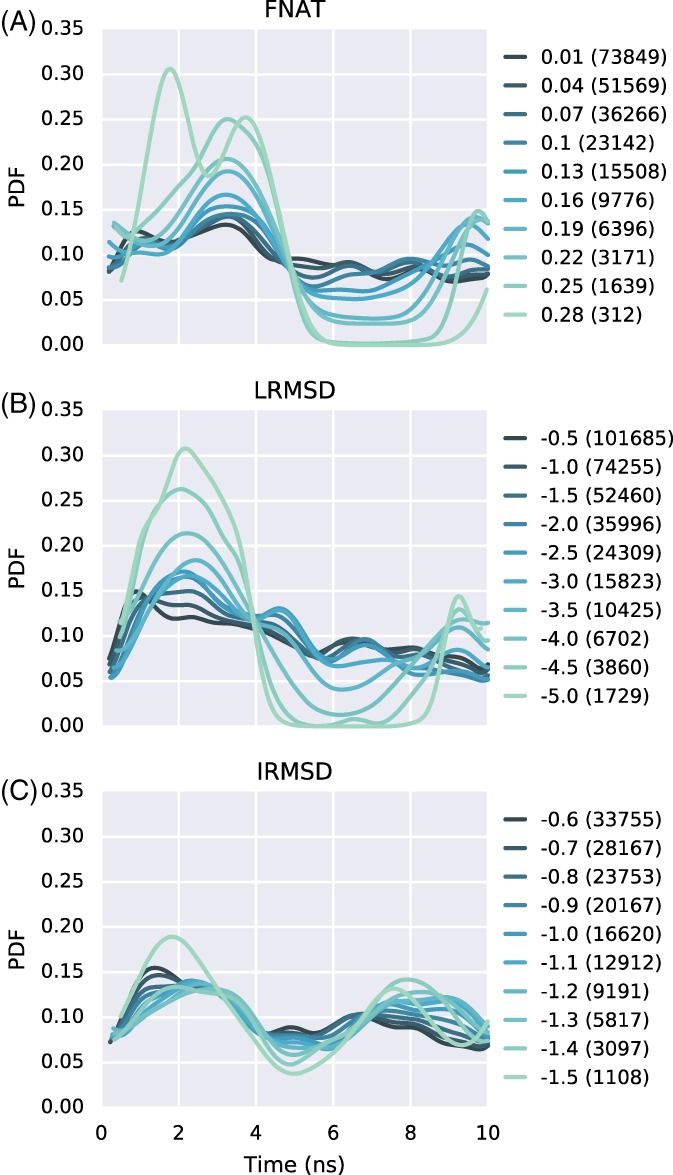
Complex refinement improvements as a function of time. Shown are the probability density functions (PDF) for improvements over time for (A) FNAT, (B) LRMSD (Å) and (C) IRMSD (Å). The different colored lines show the used threshold. The number in brackets indicates the number of snapshots ≥ the threshold for FNAT and ≤ the threshold for LRMSD and IRMSD

Analysis of the sampling power for the 5 times replicated metadynamics runs in CMS, over 10 ns, shows that large FNAT and LRMSD improvements are mostly sampled within the first 4 ns, where snapshots with improvements of ΔFNAT > 0.25 and ΔLRMSD < − 4.5 have the highest density (see Figures 3A,B). As expected, snapshots with small FNAT improvements (range 0.01‐0.1) resemble a uniform distribution, with equal density, over the sampled time. For LRMSD, the density continuously lowers with increasing sampling time, for all analyzed thresholds (see Figure 3B). This could indicate a drift away from the near‐native conformation that is also observed for refinement simulations of protein‐monomers.^39^

Improvements for large IRMSD deviations (>−1.4 Å) follow a bimodal distribution, where the highest density of these snapshots are observed around time‐points 2 ns and 8 ns, see Figure 3C. Across the complete time period sampled, the smaller IRMSD improvements follow a uniform density distribution (thresholds −0.6 Å to −1.0 Å). This may indicate that transitions to larger IRMSD improvements will require longer simulation times. However, as discussed above, longer simulations may drift models away from their native binding funnels.

### 3.3 CS_*α*_: Combining FES and ZRANK for snapshot scoring

**Figure 4:**
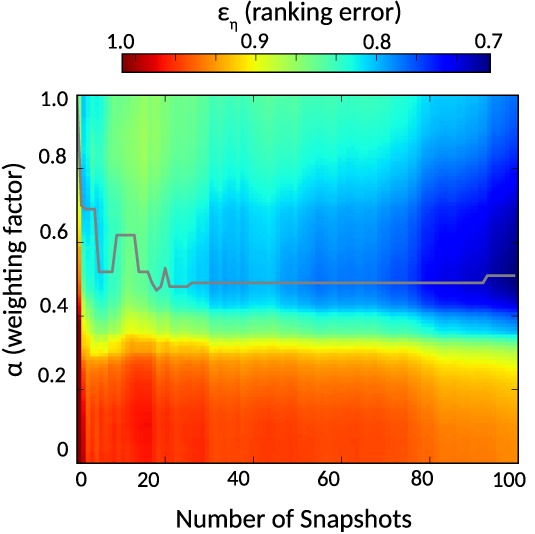
Parameter optimization of CS_*α*_ based on acceptable quality refinements. The heatmap shows the normalized ranking error *ε*_*η*_ for different *α* (y‐axis) and number of selected snapshots (x‐axis); a lower value means better. The gray line indicates the best *α*‐value for the number of snapshots, that is, for which the lowest *ε*_*η*_ was observed

We investigated whether the reconstructed FES from the metadynamics simulations gives additional benefits in selecting snapshots, by exhaustively testing, via a weighting term, *α*, how different contributions of FES and ZRANK influence the ranking error. To be precise, the effect of different *α*‐values (ranging from 0 to 1) on the snapshot ranking error, *ε*_*η*_, with respect to the number of selected snapshots *n* (ranging from 1 to 100), was explored. The heatmap in Figure 4 shows a decrease in ranking error *ε*_*η*_ when an ensemble of snapshots is selected (*n* ≥ 2) and *α* values of ≈0.5 are chosen. For example, for *n* = 35 the lowest *ε*_*η*_ with a value of 0.805 is obtained with *α* = 0.49, indicating that an almost equal contribution of ZRANK and FES is important. This is a lower ranking error compared to setting *α* = 1.0 (only ZRANK_*η*_ is considered) with *ε*_*η*_ = 0.843 and setting *α* = 0.0 (only FES_*η*_ is considered) with *ε*_*η*_ = 0.954. However, if only one snapshot is selected, that is, *n* = 1, an *α* = 1.0 (only ZRANK_*η*_ is considered) produces the lowest ranking error with *ε*_*η*_ = 0.861. Furthermore, the heatmap also shows that high contributions of FES_*η*_ and low contributions of ZRANK_*η*_, with *α* < 0.3, leads to markedly larger ranking errors.

### 3.4 Case study of T39 and T41

**Figure 5:**
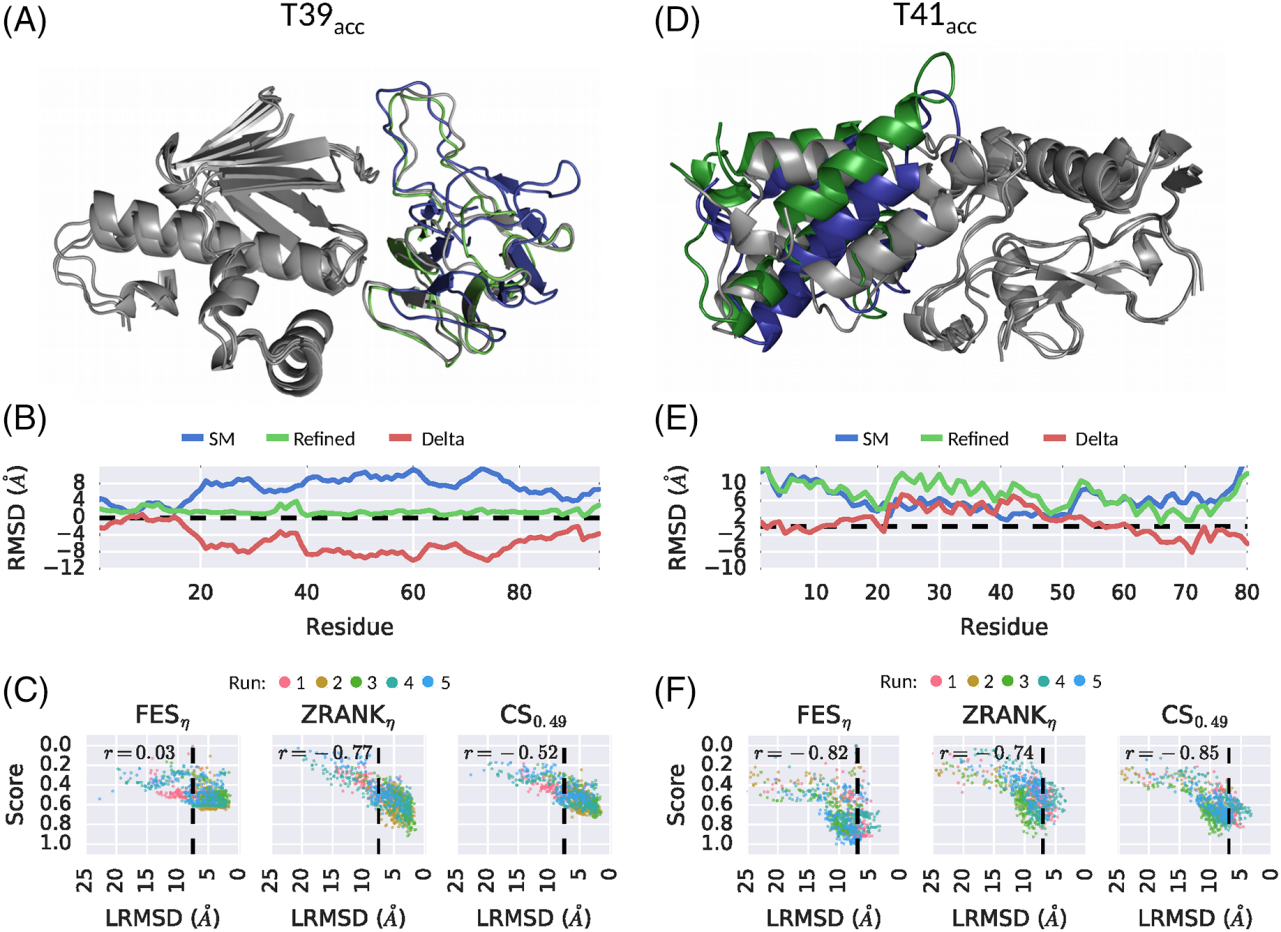
Case study of targets T39 (plots A‐C) and T41 (plots D‐F). Plots (A) and (D) 3D rendering of the protein‐ligand complex of the crystal structure (gray), starting model (SM) with acceptable quality (blue), and refined model with AZRANK and *n* = 14 (green). The starting and refined models were superimposed to the crystal structure using the receptor C*α*‐atoms. Plots (B) and (E) Per residue change of RMSD for the ligand, shown are the values for the SM (blue), refined model (green), and the difference, that is, delta, between these two (red). (C) and (F) The normalized score of FES_*η*_, ZRANK_*η*_, and CS_0.49_ for all snapshots of the refinement simulation with Δ*t* = 50ps runs 1‐5 and their correlation to LRMSD. The dotted black line indicates the LRMSD of the starting model

The 3D rendering of our refined model for target T39, built using the AZRANK strategy (setting *n* = 14), is shown in Figure 5A. This target represents the interaction of Kinesin‐like protein KIF13B (receptor) with Centaurin‐alpha‐1 (ligand).^40^ The difficulty of this target, before refinement classified as an acceptable model (T39_acc_), is associated mainly with the ligand's flexible loop regions. The results of our refinement methodology show notable success at improving the accuracy of these flexible loop regions at the protein‐protein interface. Indeed, the per‐residue change in RMSD for the ligand before and after refinement shows a continuous decrease for the whole chain. The improvements can be more than 8 Å, as indicated by the red line in Figure 5B. The scoring of the different snapshots with FES_*η*_, ZRANK_*η*_ and CS_0.49_ vs LRMSD is shown in Figure 5C. The left plot shows that FES_*η*_ has a broad energy funnel (*r* = 0.03), where snapshots with a wide range of LRMSD values (20‐1 Å) have similar energies; therefore, making a selection of the best snapshots hard for this particular target. Energy funnels associated with ZRANK_*η*_ and *CS*_0.49_ show a better correlation with LRMSD, with *r* =  − 0.77 and *r* =  − 0.52, respectively, which indicate a better fit for selecting snapshots with improvements.

An improvement in model quality was not possible for refinement target T41, starting from an acceptable quality model (see 3D rendering in Figure 5D). This target is an X‐ray structure of a complex formed between colicin E9 deoxyribonuclease (receptor) and colicin E2 immunity protein (ligand).^41^ The bound complex is characterized by an *α*‐helix (receptor) and loop interactions at the interface. The starting model, seen in blue, has large displacements, especially for the alpha‐helix, with a per‐residue RMSD of ≈3 Å to 8 Å (see residues 30‐60 in Figure 5E). After refinement with AZRANK (*n* = 14), these interface regions decreased in quality (see Figure 5D green colored rendering). A displacement with a delta change of up to 7 Å is observed (see red line in Figure 5E). Analysis of the snapshot scoring with ZRANK_*η*_, see Figure 5F, reveals a false energy‐minima that incorrectly identifies solutions with a higher LRMSD instead of the snapshots that represent a real improvement in LRMSD, thus explaining the failed model building of AZRANK. This problem is not unique to ZRANK_*η*_, function FES_*η*_ is also not able to correctly identify improved LRMSD snapshots. However, the correlation to LRMSD is substantially higher, with *r* =  − 0.82, compared to ZRANK_*η*_, with *r* =  − 0.74. The scoring function CS_0.49_, a combination of FES_*η*_ and ZRANK_*η*_, shows an even higher correlation with *r* =  − 0.85, suggesting the positive impact of combining the two functions for this particularly difficult target.

## 4 DISCUSSION

Our primary result is that metadynamics sampling in CMS yields improved quality snapshots for all targets and starting model categories. Sampled improvements for FNAT ranged from 0.01 to 0.35, for LRMSD from −0.07 Å to −6.03 Å and for IRMSD from −0.01 Å to −1.76 Å.

The new methodology is drawing on the input space of inter residue‐residue contacts, originating from an ensemble of docking poses obtained from the score_set data set. Our sampling method will bias towards this space, and indeed, as demonstrated in this study, capable of producing an even better model than any within the original ensemble. However, there is no guarantee that this will always be the case. One current limitation of our methodology is that it only incorporates potential interface residue contacts sampled by the docking community. These could be incorrect, and alternative residue‐residue contact predictions, based for example on evolutionary information^42^ that can easily be introduced into our CM_*if*_ definition, may better guide the refinement in the direction of the correct binding pose. Use of such predictions may be especially important for the more difficult docking cases, for example, where model building by homology is required to obtain one or more unbound components.

Interestingly, the largest improvements for FNAT and LRMSD were mainly sampled in the first 4 ns of the refinement runs, suggesting that, in general, shorter and more replicated runs lead to enhanced sampling power for those two metrics. An explanation for this finding is the observation that during the sampling runs disassociations between the receptor and ligand can occur, resulting in solutions with high LRMSD and low FNAT. As to why longer simulations may exhibit these observed drifts, irrespective of our constraining CMS potential, is still unexplored. It could be that some binding funnels are shallow; therefore, irrespective of the number of starting conformations found by the docking community to be approximately in the correct position, longer simulations, may have the tendency to drift away from the native binding site. Conversely, docking ensembles may already be within a deep binding funnel, and therefore, the collective variable calculated, as described in our refinement protocol, may rapidly direct the refinement toward the bottom of the binding funnel. Conversely, reliance on shorter simulation runs cannot be absolute as the free‐energy surface can be very jagged in the vicinity of the native conformation requiring longer simulations, or stronger biasing potentials than the CMS potential used here, for scaling larger energy barriers.

The main focus of the presented method was to improve directed sampling at the interface level. The metric IRMSD quantifies the conformational difference at the interface between the predicted model and the reference crystal structure state. Table 1 shows the IRMSD of used starting models, where values range from 0.80 Å to 6.12 Å. The results in column “Best Snapshot” of Table 2 and Figure 3 show that substantial improvements could be sampled for a number of targets. For example, the best snapshot for T29 improved with a ΔIRMSD of −1.59 Å from an initial value of 3.41 Å. However, sampling full transitions, where IRMSD values below 1 Å are obtained, remains challenging; this has already been observed in a previous protein complex refinement study,^19^ where all tested sampling methods failed to fully sample the full transition from an unbound to bound conformational state. The results for model building based on AZRANK, with 14 snapshots, has shown some degree of success for starting models with acceptable quality, where the FNAT, LRMSD, and IRMSD could be improved 7, 6, and 8 times out of 11 targets. If model building success is defined as improving at least one metric for each target, a success‐rate of 82% could be achieved, where 9 out of 11 targets are improved.

The described method shows potential to guide models within, or on the edge, of the binding funnel to descend the funnel and thereby enabling higher quality models to be sampled. Other refinement methods have shown similar measures of success using quite different strategies. For example, in,^43^ a general quadratic function is constructed to underestimate a set of local minima in the context of a wider scope of binding funnel. Another study facilitated refinement by using a gradual energy landscape smoothing of the binding funnel; achieved by changing the grid size resolution for docking structures in the context of the FFT docking algorithm GRAMM.^44^ However, no one method can as yet provide a complete solution to the refinement problem, as not only further improvements in modeling energy functions are required, but also the development of new algorithms to enable sufficient sampling of conformational space; for example, large to medium backbone motions between the unbound and bound conformational states are problematic for any current simulation methodology to replicate.^19^

Disappointingly, we show that ranking just on free energy, FES_*η*_ alone, produces a higher ranking error of snapshots compared to ZRANK_*η*_, and is therefore not recommended as a viable alternative for snapshot selection. However, there is some encouragement in that FES_*η*_, when combined with ZRANK_*η*_, can lead to a lower ranking error *ε*_*η*_ as shown in our analysis of CS_*α*_. The model building performance of this function is at least comparable to ZRANK_*η*_ for improving acceptable quality models. However, CS_*α*_ falls behind when medium quality or high‐quality models are refined. Thus, snapshot selection solely based on ZRANK_*η*_ is currently recommended for ranking when refinement of models of unknown quality is performed.

Importantly, as also recently discussed by several groups in the protein docking field,^45^ our results underline that the identification of improved quality snapshots, from thousands of solutions, remains one of the most challenging tasks for successful protein‐protein complex refinement. The explored combination of FES and ZRANK in a simple weighted additive scoring function, CS_*α*_, although showing some encouraging results, did not yield significant success at improving this outcome. A possible avenue to improve the blending of energy functions, to reduce snapshot ranking error, could be to use machine‐learning based scoring schemes that are able to combine different functions in a nonlinear fashion.

Such scoring schemes, based on support vector machines^46^ or extremely randomized trees,^47^ have been proposed for the global identification of correct docked models.^48^, ^49^ However, using machine learning for the specific purpose of refining local decoys, within or close to the native funnel, are to the authors knowledge, not as yet developed. A promising movement in this direction might be the identification of improved quality snapshots from refinement trajectories of protein folds,^50^ which explicitly take the temporal component of the dynamic trajectory into consideration by the use of temporal learning with deep recurrent neural networks;^51^ a methodology which can be readily adopted to protein‐protein complex refinement and selection.

## AUTHOR CONTRIBUTIONS

E.P. and P.A.B. designed the research and wrote the manuscript. E.P conducted the experiments and analyzed the data.

## CONFLICT OF INTERESTS

The authors declare no competing financial interests.

## Supporting information


**Appendix S1: Supporting Information**
