# Supplementary material for: Refinement of protein‐protein complexes in contact map space with metadynamics simulations
Source: Proteins. 2018 Oct 30;87(1):12–22. doi: 10.1002/prot.25612 (PMC6492248; doi:10.1002/prot.25612)
Supplement: Supplementary file 1 — Appendix S1: Supporting Information [file PROT-87-12-s001.pdf]

Supplemental Material

Optimizing for the Number of Snapshots

A detailed visualisation of the effect of the number of selected snapshots n on model building is shown in Figure S1 and S2 which show the results as aggregated averages for each starting model quality and each individual data point, respectively. Function ZRANK_{η} is able to produce, on average, improved models for starting models with acceptable quality, and over a wide range of n (Figure S1, left columns and blue labels). Where the best results for average ΔFNAT, ΔLRMSD and ΔIRMSD was achieved at $n = 14$ with 0.07, $n = 16$ with -1.16 Å and $n = 61$ with -0.41 Å, respectively. For medium quality starting models (Figure S1, left columns and green labels) the average quality decreased with a value of ΔFNAT= -0.01 ($n = 18$) and increased slightly with ΔLRMSD=-0.25 Å ($n = 189$) and ΔIRMSD=-0.18 Å ($n = 193$). As expected, on average, high quality starting models could not be improved. For this category, all three metrics decreased in quality after refinement (left columns and red labels). The best values were obtained for FNAT, IRMSD and LRMSD at $n = 7$ with -0.11, $n = 7$ with 1.74 Å and $n = 109$ with 0.59 Å, respectively.

The Center columns of Figure S1 show the results for FES_{η}. The average ΔFNAT for acceptable, medium and high starting models could not be improved with this scoring function and yielded the best results at $n = 196$ with -0.10, $n = 73$ with -0.10 and $n = 70$ with -0.34, respectively (Figure S1a). For ΔLRMSD, only acceptable models could be improved where the best $n = 193$ had a ΔLRMSD of -0.39 Å. Medium and high quality models decreased on average in quality by 0.96 Å ($n = 129$) and 3.82 Å ($n = 70$), respectively (Figure S1b). Similar results are observed for ΔIRMSD, as shown in Figure S1c, where only acceptable starting models could be improved by -0.486 Å ($n = 189$). The ΔIRMSD increased for medium and high by 0.01 Å ($n = 191$) and 1.25 Å ($n = 70$).

The function $CS_{0.49}$, shown in the right columns of Figure S1, did not improve model building performance compared to ZRANK_{η}, and is only on par for acceptable starting models. Here, the best average ΔFNAT, ΔLRMSD and ΔIRMSD improvement was yield at $n = 20$ with 0.06, $n = 38$ with -1.11 Å and $n = 93$ with -0.38 Å, respectively. For medium quality starting models, the ΔFNAT decreased at best $n = 162$ with -0.05 and improved for ΔLRMSD and ΔIRMSD for best $n = 135$ with -0.06 Å and $n = 194$ with -0.14 Å, respectively. Finally, for high quality models all three metrics ΔFNAT, ΔLRMSD and ΔIRMSD decreased after refinement for all n tested snapshots. The smallest decrease in quality was obtained at $n = 146$ with -0.15 (FNAT), $n = 188$ with 2.45 Å (LRMSD) and $n = 188$ with 0.77 Å (IRMSD), respectively.

Missing Atom and Gap Modeling with SCWRL and Loopy

Missing residue side-chain atoms in a PDB structure were modeled with SCWRL. The affected residues were marked in the SCWRL sequence input file as flexible, all other residues were marked as fixed side-chains, thus, are left untouched. Missing intra-chain residues in a PDB structure are modeled with Loopy. Gaps are determined from a sequence alignment with a provided reference amino-acid sequence of the protein and the

extracted amino-acid sequence from the PDB file. Loopy is run with CHARMM22 force field parameters in fast mode, where 1000 initial conformations are generated.

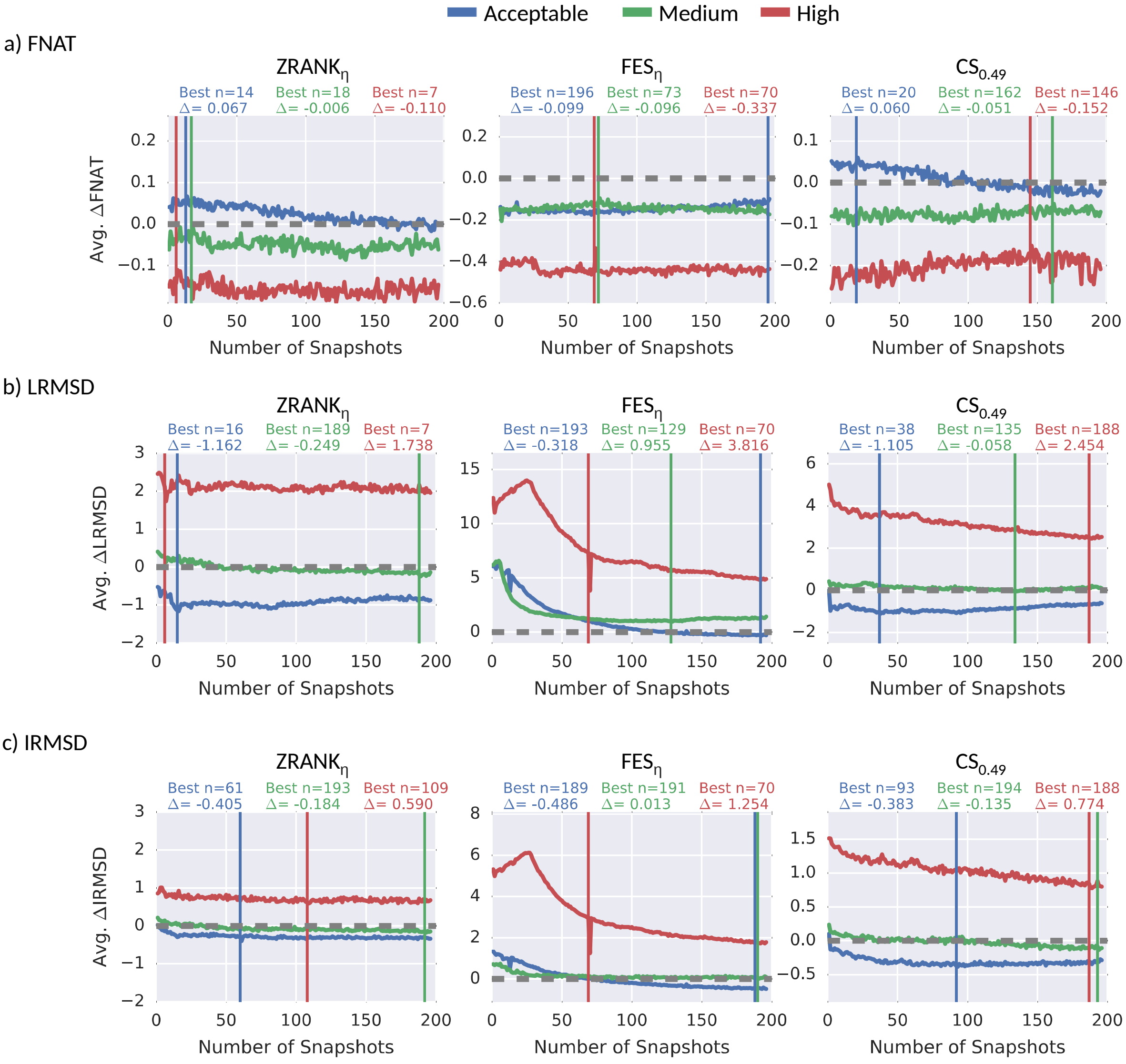

Figure S1 : Average Δ-changes as a function of number of snapshots for build models with AZRANK. a) ΔFNAT b) ΔLRMSD (Å), c) ΔIRMSD (Å). The three colors blue, green and red represent the target's starting model quality category, i.e., acceptable, medium and high, respectively. The grey dotted line indicates the Δ-value at 0.0. The three lines for each subplot a,b and c show the average value grouped by the three different starting model categories. The vertical lines indicate the best n, based on best average performance for the metric; values of best performing n and average Δ-change are shown above each plot.

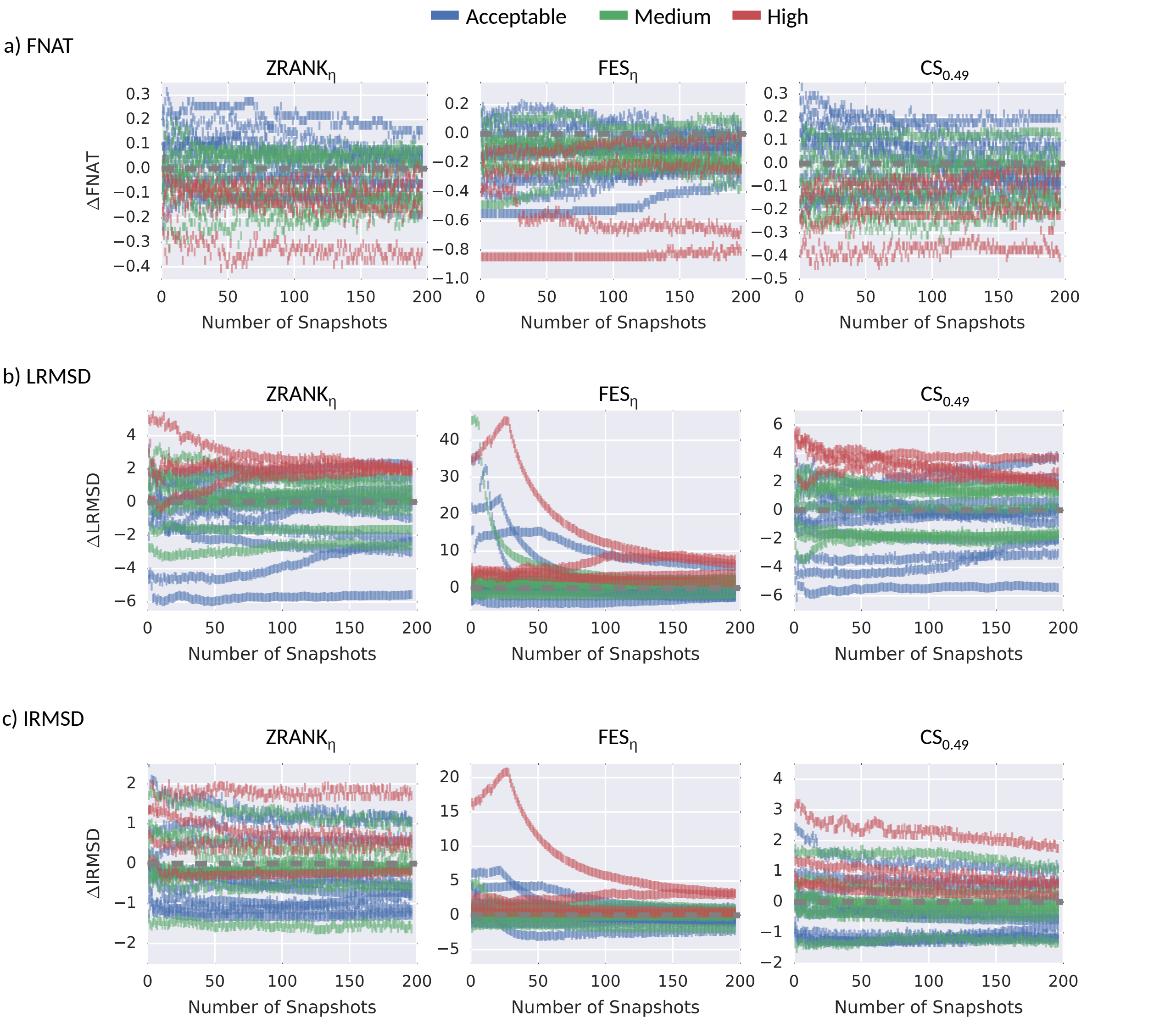

Figure S2 : Individual Δ-changes as a function of number of snapshots for each build model with AZRANK for all benchmark targets . a) ΔFNAT b) ΔLRMSD (Å), c) ΔIRMSD (Å). The three colors blue, green and red represent the target's starting model quality acceptable, medium and high, respectively.
